# Reduced intensity of early intensification does not increase the risk of relapse in children with standard risk acute lymphoblastic leukemia - a multi-centric clinical study of GD-2008-ALL protocol

**DOI:** 10.1186/s12885-020-07752-x

**Published:** 2021-01-13

**Authors:** Xin-Yu Li, Jia-Qiang Li, Xue-Qun Luo, Xue-Dong Wu, Xin Sun, Hong-Gui Xu, Chang-Gang Li, Ri-Yang Liu, Xiao-Fei Sun, Hui-Qin Chen, Yu-Deng Lin, Chi-kong LI, Jian-Pei Fang

**Affiliations:** 1 Department of Pediatrics, Sun Yat-sen Memorial Hospital, Sun Yat-sen University, Guangzhou, 510120 China; 2 Guangdong Provincial Key Laboratory of Malignant Tumor Epigenetics and Gene Regulation, Sun Yat-sen Memorial Hospital, Sun Yat-sen University, Guangzhou, 510120 China; 3 The First Affiliated Hospital, Sun Yat-sen University, Guangzhou, 510080 China; 4 Nanfang Hospital, Southern Medical University, Guangzhou, 510515 China; 5 Guangzhou Women and Children’s Medical Center, Guangzhou, 510623 China; 6 Shenzhen Children’s Hospital, Shenzhen, 518038 China; 7 Huizhou Municipal Central People’s Hospital, Huizhou, 516001 China; 8 Sun Yat-sen University Cancer Center, Guangzhou, 510060 China; 9 The Third Affiliated Hospital, Sun Yat-sen University, Guangzhou, 510630 China; 10 Guangdong General Hospital, Guangzhou, 510080 China; 11 Hong Kong Children Hospital and Prince of Wales Hospital, The Chinese University of Hong Kong, Hong Kong, 999077 China

**Keywords:** Acute lymphoblastic leukemia, Children, Chemotherapy, Reduced intensity multi-centric clinical study

## Abstract

**Background:**

The prognosis of childhood acute lymphoblastic leukemia (ALL) is optimistic with a 5-year event-free survival (EFS) rate of 70–85%. However, the major causes of mortality are chemotherapy toxicity, infection and relapse. The Guangdong (GD)-2008-ALL collaborative protocol was carried out to study the effect of reduced intensity on treatment related mortality (TRM) based on Berlin-Frankfurt-Münster (BFM) 2002 backbone treatment. The study was designed to elucidate whether the reduced intensity is effective and safe for children with ALL.

**Methods:**

The clinical data were obtained from February 28, 2008 to June 30, 2016. A total of 1765 childhood ALL cases from 9 medical centers were collected and data were retrospectively analyzed. Patients were stratified into 3 groups according to bone marrow morphology, prednisone response, age, genotype, and karyotype information: standard risk (SR), intermediate risk (IR) and high risk (HR). For SR group, daunorubicin was decreased in induction IA while duration was reduced in Induction Ib (2 weeks in place of 4 weeks). Doses for CAM were same in all risk groups - SR patients received one CAM, others got two CAMs.

**Results:**

The 5-year and 8-year overall survival (OS), event-free survival (EFS) and cumulative incidence of relapse (CIR) were 83.5±0.9% and 83.1±1.0%, 71.9±1.1% and 70.9±1.2%, and 19.5±1.0% and 20.5±1.1%, respectively. The 2-year treatment-related mortality (TRM) was 5.2±0.5%. The 5-year and 8-year OS were 90.7±1.4% and 89.6±1.6% in the SR group, while the 5-year and 8-year EFS were 81.5±1.8% and 80.0±2.0%. In the SR group, 74 (15.2%) patients measured minimal residual disease (MRD) on Day 15 and Day 33 of induction therapy. Among them, 7 patients (9.46%) were MRD positive (≥ 0.01%) on Day 33. The incidence of relapse in the MRD Day 33 positive group (*n*=7) was 28.6%, while in the MRD Day 33 negative group (*n*=67) was 7.5% (*p*=0.129).

**Conclusions:**

The results of GD-2008-ALL protocol are outstanding for reducing TRM in childhood ALL in China with excellent long term EFS. This protocol provided the evidence for further reducing intensity of induction therapy in the SR group according to the risk stratification. MRD levels on Day 15 and Day 33 are appropriate indexes for stratification.

**Supplementary Information:**

The online version contains supplementary material available at 10.1186/s12885-020-07752-x.

## Background

Acute lymphoblastic leukemia (ALL) is the most common malignant disease of children. In recent decades, with the improvement of diagnosis, classification and stratified treatment in China, the disease-free survival rate has gradually improved. The 5-year event-free survival (EFS) rate of children with ALL has reached 70–80% in multi-center studies, and even 85% in some studies [1–12]. However, the major causes of mortality are chemotherapy toxicity, infection and relapse; infections result from bone marrow toxicity. In earlier decades in mainland China, reduced intensity in ALL chemotherapy was studied by pediatricians by performing serial protocol modifications which tremendously reduced chemotherapy-related mortality. The GD-2008-ALL collaborative protocol was one of them.

Nowadays, with the clinical practice of measuring minimal residual disease (MRD) in leukemia, risk stratification has been improved [13–15]. The MRD levels after remission induction therapy is a more dependable tool for prediction of relapse and has helped determine adjustments for intensity for chemotherapy [13–15]. Further reducing intensity of chemotherapy based on MRD has been an appealing area of study.

By retrospectively analyzing the clinical data of the GD-2008-ALL collaborative group, this report evaluated the efficacy and safety of the overall and risk classification treatment in GD-2008-ALL protocol in the past 10 years. The study of GD-2008-ALL protocol was designed to reduce the intensity of induction therapy for children aiming at reduction of therapy related mortality (TRM) based on the Berlin-Frankfurt-Münster (BFM) ALL IC-BFM 2002 standard treatment backbone [3, 5] and to elucidate whether the reduced dosage and duration of early intensification based on the multifactor basing risk stratification is effective and safe for the standard risk (SR) group and the intermediate risk (IR) group. In this report, we will find data evidence for reducing dose of cyclophosphamide (CTX), Cytarabine (Ara-c) and 6-mercaptopurine (6-MP) during induction for the SR group based on the MRD level on Day 33.

## Methods

### Patients

From 28th February, 2008 to 28th June, 2016, 1765 children (1-18 years old) who were with newly diagnosed ALL underwent chemotherapy according to the GD-2008-ALL protocol in nine collaborative centers as follows: Sun Yat-sen Memorial Hospital of Sun Yat-sen University (*n*=410), Guangzhou Women and Children Medical Center (*n*=331), First Affiliated Hospital of Sun Yat-sen University (*n*=296), Southern Medical University Affiliated Southern Hospital (*n*=282), Shenzhen Children’s Hospital (*n*=269), Huizhou Central People’s Hospital (*n*=72), Third Affiliated Hospital of Sun Yat-sen University (*n*=49), Sun Yat-sen University Cancer Center (*n*=50), Guangdong People’s Hospital (*n*=6). Followed up was until 30 June 2018. The median follow-up was 4.9 years (range, 0 to 8 years).

All inclusion and exclusion criteria are listed as follows: inclusion criteria: (1) first-time diagnosed with ALL; (2) the age at disease onset was 1–18 years old; (3) the guardian signed the informed consent and participated in the GD-2008-ALL regimen for chemotherapy; exclusion criteria: (1) ALL was the secondary tumor or relapsed ALL; (2) ALL was a definite CML transformation; (3) Down’s syndrome; (4) mature B lymphocytic leukemia/lymphoma; (5) previous chemotherapy before admission (including glucocorticoid for more than 1 week); (6) risk classification of chemotherapy data were incomplete;(7) unable to finished 80% of the total doses of regimen; (8) patients quit the study. Patients who finished less than 80% of the total doses because of fatal side effects were included for analysis. The research protocol and informed consent were approved by the ethics committee of Sun Yat-sen Memorial Hospital of Sun Yat-sen University and cooperation agreements were signed with all collaborative members. All collected data were retrospectively analyzed.

### Diagnosis

The GD-2008-ALL protocol diagnosed patients according to cell morphology, immunology, cytogenetics, and molecular biology (MICM) classification. ALL was diagnosed if at least 25% lymphoblasts were present in bone marrow (BM). Each collaborative center finished morphology diagnosis independently. If the morphology results were disputed, bone marrow slices were sent to the protocol laboratory, including the hematology labs of Kingmed Diagnostics Coorperation and Kindstar Global Coorperation, which were certificated by the International Organization for Standardization (ISO 15189), for review. Immunophenotyping was performed according to European Group for the Immunological Characterization of Leukemias (EGIL) or World Health Organization (WHO) 2008 criteria [16–18]. Immunity classification criteria were in accordance with previous literature and are provided in the Supplementary information (SI 1) [16, 17]. Mature B lymphocyte leukemia and infantile leukemia were excluded from the cohort. Karyotyping and molecular genetics (fluorescent in situ hybridization [FISH], multiplex nester reverse transcriptase (PCR) to investigate BCR-ABL and mixed lineage leukemia (MLL)-AF4 were mandatory. Criteria for central nervous system (CNS) involvement and CNS relapse are listed in the Supplementary information (SI 2.). Patients were registered at the protocol data management office within 24 to 72 h after the start of prednisone treatment.

### Treatment response and relapse criteria

Prednisone response was determined by absolute blast count in peripheral blood on Day 8, after 7 days of prednisone and one dose of intrathecal methotrexate (MTX) on Day 1. Prednisone poor response (PPR) was defined as ≥ 1 X 10^9^/L blasts, and prednisone good response (PGR) was defined as less than 1 X 10^9^/L blasts. BM response to induction therapy was evaluated by morphology on Day 15 and Day 33. Complete remission (CR) was defined as less than 5% blasts in a regenerating marrow on Day 33 and no extramedullary disease. Failure to achieve CR by Day 33 was not considered an event and was only triaged to HR. Resistance to therapy (nonresponse) was defined as no CR by the start of the third consolidation HR block. BM relapse was defined as reappearance of 25% lymphoblasts in BM. Combined relapses meant recurrence in both BM and extramedullary site(s). TRM is death which occurs during chemotherapy without recurrence or secondary tumor [3, 5].

### Stratification

**Fig. 1:**
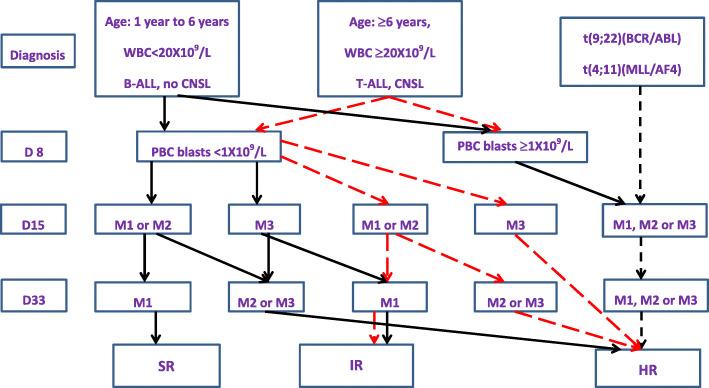
Risk classification criteria of GD-2008-ALL chemotherapy regimen. The process of patient stratification into 3 groups: standard risk (SR), intermediate risk (IR) and high risk (HR). M1 (< 5% blasts), M2 (5% to ≤25% blasts),M3 (> 25% blasts)

Patients were stratified into 3 groups - SR, IR and high risk (HR) - according to the age of onset, peripheral blood leukocyte counts since onset, immune classification (B ALL or T ALL), fusion genes, central nervous system leukemia (CNSL), testicular lymphoblast invasion, mediastinal invasion and the response to 7-day prednisone treatment and remission on Day 15 and Day 33 (Fig. 1).

SR is defined as PGR, age 1 year to younger than 6 years, initial white blood cell count (WBC) less than 20 X 10^9^/L, and M1 (< 5% blasts) or M2 (5% to ≤25% blasts) marrow on Day 15 of induction chemotherapy, and M1 bone marrow on Day 33 (all criteria fulfilled). IR is defined as PGR, age 6 years or older, and/or WBC ≥ 20 X 10^9^/L and M1 or M2 marrow on Day 15 and M1 marrow on Day 33, or SR criteria fulfilled but M3 (> 25% blasts) marrow on Day 15 and M1 marrow on Day 33, or CNS-3 at the first diagnosis without HR criteria fulfilled, or T-ALL without HR criteria fulfilled.

HR is defined as at least one of the following: PPR, IR and M3 marrow on Day 15, M2 or M3 marrow on Day 33, t(9;22) (BCR-ABL), or t(4;11) (MLL-AF4), or testicular leukemia suspected at onset and not relieved at end of induction (confirmed by testicular biopsy), or residual mediastinal mass in the first week after the end of induction.

### Minimal residual disease evaluation

Flow cytometry (FCM)-MRD was analyzed according to previous literature from French multicenter study groups for pediatric and adult ALL [19, 20]. MRD was analyzed at the central protocol laboratory- the hematology labs of Kingmed Diagnostics Coorperation by Kaluza software or Cellquest software. Reagents were provided from BD Biosciences (Becton, Dickinson, China) and Beckman Coulter Commercial Enterprise (China) Co., Ltd. MRD Day15 positive was defined as MRD ≥0.1%, while MRD Day 33 positive was defined as MRD≥0.01%.

### Treatment and toxicity

The treatment outline is depicted in Fig. 1. Details of chemotherapy are provided in Table 1; details of intrathecal injection (IT) medication and dose are provided in the supplementary information (SI 3. Table S1); the cumulative doses of medicine in induction and reinduction therapy are provided in the supplementary information (SI 4. Table S2). Compared with the BFM-2002 therapy backbone, there are modifications in CAM chemotherapy: (1) the induction CAM chemotherapy in the SR group was reduced to 2 weeks; (2) the 4-week CAM chemotherapy was cut in the middle by a 2-week interruption when hematopoiesis recovery was allowed in the IR group. In SR patients with B-cell precursor ALL (BCP-ALL), two doses of daunorubicin were prescribed in induction compared with four doses in all others. In consolidation, high-dose methotrexate was administered at 5 g/m^2^ for SR/IR T-ALL and at 2 g/m^2^ for SR/IR BCP-ALL. Consolidation for HR patients consisted of six intensive polychemotherapy blocks. A single re-induction protocol (VDLD3/VDLD4 plus CAM2) was given in the SR/IR/HR groups. Prophylactic cranial radiotherapy was not recommended. Therapeutic cranial radiotherapy was reserved for patients with initial CNS involvement and given at an age-adjusted dosage: 12 Gy for children age 1 to younger than 2 years, and 18 Gy for children age 2 years and older.

**Table 1:** GD-2008-ALL protocol

treatment stages	SR group	IR group	HR group
Induction IA	Prednisone test	Prednisone test	Prednisone test
	VDLD1	VDLD2	VDLD2
Induction Ib	CAM	CAM	CAM
		14 days interval	14 days interval
		CAM	CAM
Consolidation	mM	mM for B-ALL/M for T-ALL	(HR-1′,HR-2′,HR-3′)X2
Re-induction	VDLD3	VDLD3	VDLD4
	CAM2	CAM2	CAM2
maintenance	A or B	A or B	A or B

Note:

Induction IA: Prednisone test and VDLD1/ VDLD2

Induction IB: one CAM in SR group; two CAM in IR/HR groups

Prednisone test: d1-d7: prednisone 60 mg/(m^2^·d), taken orally in three times (gradually increase from 15 mg/(m^2^·d) to the full dose according to clinical response. The cumulative dose should be more than 210 mg/m^2^ in 7 days. For patients with high tumor load, the initial dose can be reduced by 0.2–0.5 mg/(m^2^·d) to avoid tumor lysis syndrome)

VDLD1: Dexamethasone (DEX, day 8 to 28, 6 mg/(m^2^·d), divided into three times; day 29 to 31, 3 mg/(m^2^·d); day 32–34 1.5 mg/(m^2^·d); day 35–37 0.75 mg/(m^2^·d). Vincristine (VCR): 1.5 mg/(m^2^·d) (≯ 2 mg/day), intravenous injection, d8, d15, d22, d29.Daunorubicin (DNR): 30 mg/(m^2^·d), intravenous, on day 8 and day 15.L-asp: 5000 U/(m^2^·d), intravenous infusion, day 12, day 15, day 18, day 24, day 27, day 30, day 33(8 times in total). Intrathecal therapy (IT): day 1, day 15,day 33

VDLD2: extra two doses of DNR in VDLD1 30 mg/(m^2^·d), on day 22 and day 29

CAM: cyclophosphamide (CTX): 1000 mg/(m^2^·d), PI>lh, day 1; 6-mercaptopurine (6-MP): 60 mg/(m^2^·d), day 1 to day 14; Cytarabine (Ara-c): 75 mg/(m^2^·d), intravenous infusion, day 3 to day 6, day 10 to day 13; IT: day 10

mM: high-dose methotrexate (MTX), 2 g/(m^2^·d). 10% of the total dose is infused in the first 30 min, and the rest 90% of the total dose is infused continuously in the next 23.5 h on day 8, day 22, day 36 and day 50 of the consolidation therapy; 6-MP, 25 mg/(m^2^·d), orally, day 1 to day 56; IT: in the first 2 h after each intravenous infusion of MTX

M: high-dose methotrexate (MTX), 5 g/(m^2^·d) (for T-ALL only), 10% of the total dose is infused in the first 30 min, and the rest 90% of the total dose is infused continuously in the next 23.5 h on day 8, day 22, day 36 and day 50 of the consolidation therapy; 6-MP, 25 mg/(m^2^·d), orally, day 1 to day 56; IT: in the first 2 h after each intravenous infusion of MTX

VDLD3: DEX, day 1 to day 21, 8 mg/(m^2^·d), divided into three times; day 22 to 24, 4 mg/(m^2^·d); day 25–27 2 mg/(m^2^·d); day 28–30 1 mg/(m^2^·d). VCR: 1.5 mg/(m^2^·d) (≯ 2 mg/day), intravenous injection, d8, d15, d22, d29. Adriamycin (DOX): 30 mg/(m^2^·d), intravenous infusion, on day 8, day 15, day 22 and day 29.L-asp: 10000 U/(m^2^·d), intravenous infusion, day 8, day 11, day 15, day 18 (4 times in total). IT: day 1, day 15,day 33

CAM2: CAM plus IT on day 3

Maintenance A:6-MP:50 mg/m^2^ daily and MTX 20 mg/m^2^ once a week for continuous 74 weeks; IT: once in week 4, week 8, week 12 and week 16(T-ALL, extra IT in week 20 and week)

Maintenance B: 6-MP:50 mg/m^2^ daily and MTX 20 mg/m^2^ once a week for continuous 7 weeks, followed with VCR:1.5 mg/m^2^, intravenous injection once and DEX 6 mg/m^2^ daily for 1 week. This eight-week protocol repeats for 9 times. 6-MP:50 mg/m^2^ daily and MTX 20 mg/m^2^ once a week for continuous 2 weeks; IT:the same to omaintenance A。

HR-1′:DEX 20 mg/(m^2^·d), iv day 1 to day 5; VCR 1.5 mg/m^2^ iv d1, d6; MTX 5000 mg/m^2^, iv, PI:24 h, d1; CTX 200 mg/m^2^, iv,every 12 h for 5 times, day 2 to day 4; Ara-C 2000 mg/m^2^, iv, every 12 h for 2 times, day 5; L-ASP 25000 U/m^2^ iv, day 6 and day 11; IT d1

HR-2′:DEX 20 mg/(m^2^·d), iv, day 1 to day 5; Vincristine (VDS) 3 mg/(m^2^·d) iv, day 1 and day 6; MTX 5000 mg/m^2^, iv, PI> 24 h, day 1; ifosfamide (IFO) 800 mg/m^2^ iv, PI> 1 h, every 12 h for 5 times, day 2 to day 4; L-ASP 25000 U/m^2^ iv, PI> 2 h, day 6 and day 11; DNR 30 mg/m^2^, iv, PI> 24 h, day 5; IT d1。

HR-3′: DEX 20 mg/(m^2^·d), iv, day 1 to day 5; Ara-C 2000 mg/m^2^, iv, PI> 3 h, every 12 h for 4 times, day 1 to day 2; Etoposide 100 mg/m^2^ iv, PI> 1 h, every 12 h for 5 times, day 3 to day 5; L-ASP 25000 U/m^2^ iv, PI> 2 h, day 6, day 11; IT day 5。

VDLD4: DEX 10 mg/(m^2^·d), divided into three oral doses, from day 1 to day 28; 5 mg/(m^2^·d) from day 29 to 31; 2.5 mg/(m^2^·d) from day 32–34; 1 mg/(m^2^·d) from day 35–37. VCR: 1.5 mg/(m^2^ · d) (≯ 2 mg/day), intravenous injection, day 8, day 15, day 22 and day 29. Adriamycin (DOX): 25 mg/(m^2^·d), intravenous infusion maintenance (PI)> 1 h, day 8, day 15, day 22, day 29; L-asp: 10000 U/(m^2^·d), PI> 1 h, day 8, day 11, day 15, day 18(4 times in total)

Allogeneic hematopoietic stem-cell transplantation (HSCT) from a matched sibling donor (MSD) or a matched unrelated donor was recommended for very-high-risk patients defined as no CR by Day 33; HR plus M3 on Day 15; Philadelphia chromosome-positive (Ph+) ALL; PPR plus any of T-ALL, pro-B-ALL (very early CD10+ BCP-ALL), WBC more than 100 X 10^9^/L, or t(4;11) (MLL-AF4). HSCT was performed when the third polychemotherapy block was finished if there were suitable donors.

### Statistical analysis

All patients follow-up was done by outpatient service and telephone. All patients without outpatient follow-up records within 6 months before the end of the study were confirmed by telephone follow-up. “Withdrawn” is defined as having lost contact for over 6 months after completion of treatment. EFS and overall survival (OS) were calculated from the date of diagnosis to the date of the first event. For EFS, events were resistance, relapse, death, second malignant neoplasm or last follow-up. For OS, event was mortality of any cause. If no event occurred, the observation time was censored at the last follow-up. EFS and OS curves were estimated according to Kaplan-Meier with standard error (SE) from Greenwood and compared by two-tailed log rank test. Cumulative incidence curves for events were estimated by adjusting for competing risks and were compared by Gray test. Chi square tests were used to calculate comparisons between groups. Multivariate analysis was performed using a Cox model. Toxicity was graded by modified Common Terminology Criteria for Adverse Events (CTCAE) v3.0. Follow-up was updated as of 30th June, 2018. Statistical analysis was performed by using SPSS 22.0 (SPSS Institute, Cary, NC). *P* values < 0.05 were considered significant.

## Results

**Fig. 2:**
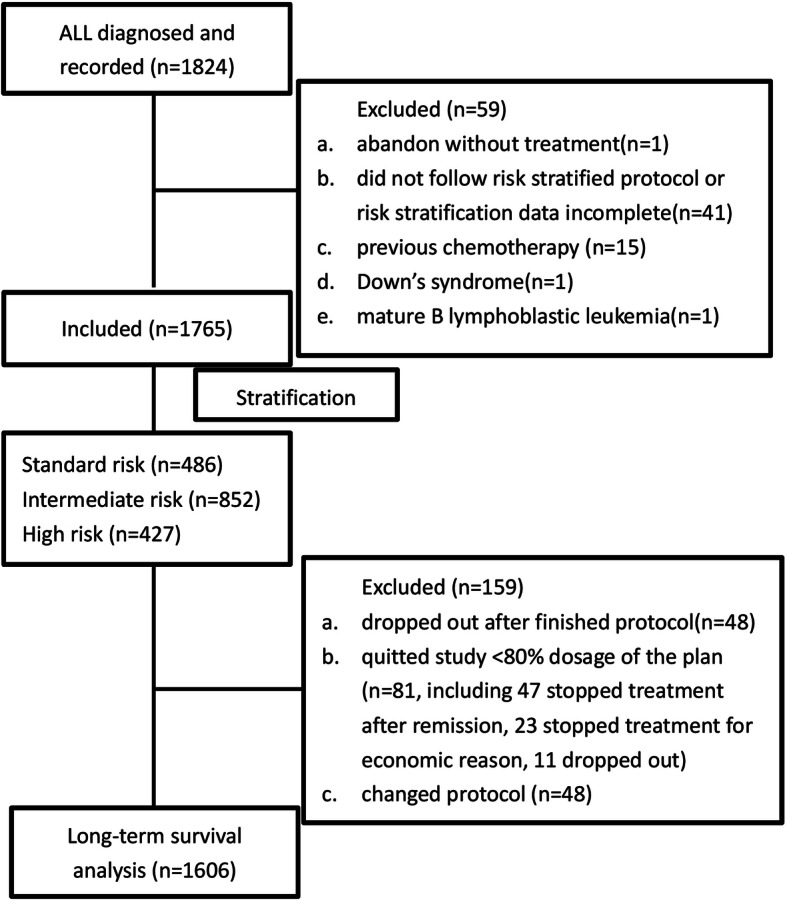
CONSORT flow diagram

**Fig. 3:**
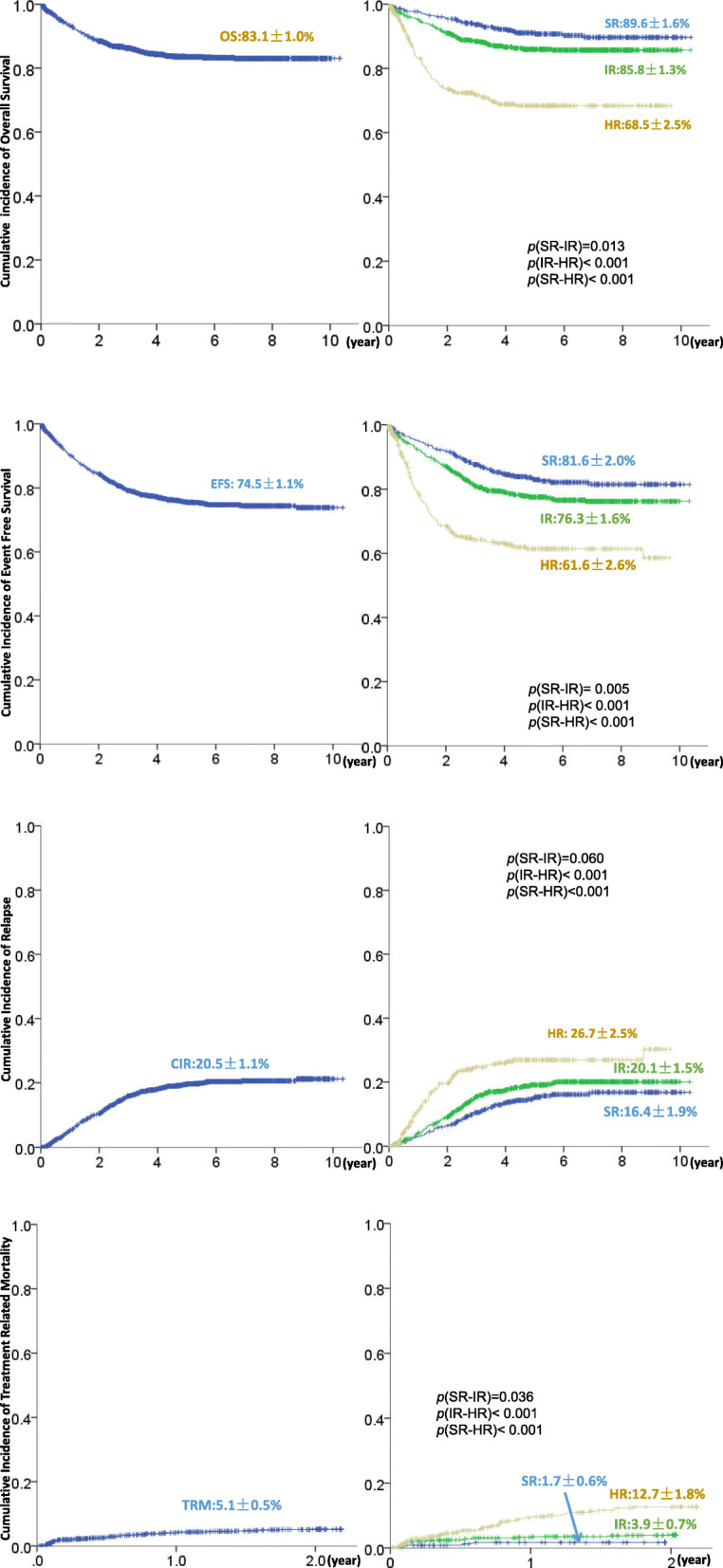
Survival curves of GD-2008-ALL study. **a** The 8-year cumulative overall survival of GD-2008-ALL protocol, SR, IR, and HR groups respectively. **b** The 8-year cumulative event free survival of GD-2008-ALL protocol, SR, IR, and HR groups respectively. **c** The 8-year cumulative incidence of relapse of GD-2008-ALL protocol, SR, IR, and HR groups respectively. **d** The 5-year cumulative incidence of treatment related mortality of GD-2008-ALL protocol, SR, IR, and HR groups respectively

One thousand seven hundred sixty-five cases of childhood ALL diagnosed and treated according to the GD-2008-ALL protocol in nine collaborative centers were included in this retrospective study. The male to female ratio was 1.72. Median age was 4.40 (range 1.0 to 17.0) years. The basic information at diagnosis is listed in Table 2. Results for prednisone response, bone marrow remission on Day 15 and Day 33 are shown in Table 3. Nine cases died of disease progression during induction chemotherapy. There were 486 cases (27.6%) stratified in the SR group, 852 cases (48.2%) in the IR group, and 427 cases (24.2%) in the HR group.1606 were included in the long-term survival analysis of GD-2008-ALL protocol. The CONSORT flow diagram is provided in the Fig. 2. The 5-year and 8-year OS were 83.5±0.9% and 83.1±1.0%, respectively. The 5-year and 8-year EFS were 75.6±1.1% and 74.5±1.1%, respectively. The 5-year and 8-year cumulative incidence of relapse (CIR) were 19.5±1.0% and 20.5±1.1%, respectively. The 2-year TRM was 5.1±0.5%. Survival curves are shown in Fig. 3. Two hundred ninety-nine cases relapsed (16.9%). The median relapse time was 20.5 months (range 1.8 to 105.0 months). Among the relapse cases, 37 (12.3%) were early relapse (< 6 months), while 53 cases (17.7%) were late relapse (> 36 months). There were 68 cases (14.0%), 146 cases (17.2%) and 85 cases (19.9%) of relapse in the SR, IR and HR groups, respectively. BM relapse occurred in 205 cases (68.6%). CNS relapse occurred in 47 cases (15.7%). Testicular relapse occurred in 21 cases (7.0%). BM and simultaneous CNS relapse occurred in 13 (4.3%). There were 7 cases (2.3%) of bone marrow plus testicular relapse and 2 cases (0.7%) of CNS plus testicular relapse. And, there were cases reported with multiple sites relapse, like 1 case of BM plus CNS plus mediastinum plus kidneys, 1 case of BM plus breasts, 1 case of BM plus mediastinum, and 1 case of pelvic cavity.

**Table 2:** The basic information at diagnosis

Subjects		N	Proportion (%)
Gender	Male	1117	63.3
	Female	648	36.7
Age (years)	<1	24	1.4
	≥1 and <6	1136	64.4
	≥6 and <10	343	19.4
	≥10	262	14.8
Immune phenotype	B	1527	88.6
	T	168	9.7
	Double phenotype	29	1.7
Fusion gene	BCR/ABL	63/1664	3.8
	MLL	77/1114	6.9
	TEL/AML1	165/1115	14.8
White blood cell counts (X 10^9^/L)	<4	383/1760	21.8
	≥4 and <10	482/1760	27.4
	≥10 and<20	271/1760	15.4
	≥20 and<50	274/1760	15.6
	≥50 and<100	150/1760	8.5
	≥100	200/1760	11.4

**Table 3:** Prednisone response, bone marrow remission on Day 15 and Day 33

Subjects		Numbers	Proportion(%)
Prednisone test	Good response	1569	88.9
	Poor response	186	10.5
	Unable to evaluate	10	0.6
Day 15 of remission	M1	1132	68.7
	M2	296	18.0
	M3	220	13.3
	NA	117	
Day 33 of remission	M1	1651	97.1
	M2	31	1.8
	M3	19	1.1
	NA	64	
Stratification	SR	486	27.5
	IR	852	48.3
	HR	427	24.2

Note: *NA* not available. M1: < 5% blasts. M2: 5% to ≤25% blasts. M3: > 25% blasts. *SR* standard risk. *IR* intermediate risk. *HR* high risk

There were 266 cases of death, with a total mortality of 15.1%. One hundred fifty-eight patients (59.2%) died within 6 months after relapse, which included 109 cases who stopped treatment after relapse, 94 patients who died from infections (35.2%), 22 patients who died from bleeding and thrombosis (8.3%), and 21 patients who died after transplantation (26.6%). There were 47 HR patients who accepted HSCT at first CR. Thirty-three of them were persistently disease-free, while 5 patients suffered relapse after transplantation. Two patients, who relapsed post-HSCT, got persistent CR2 by chemotherapy and immunotherapy. Secondary tumors were found in 4 patients, including rhabdomyosarcoma, Hodgkin’s lymphoma, acute myeloid leukemia, and T lymphoblastoma/leukemia.

The 5-year and 8-year OS rates, EFS rates, CIR and 2-year TRM of SR, IR and HR groups are listed in Table 4. In the SR group, the incidence of adverse effects, including abnormal liver transaminase, infection, and TRM were 4.7% (*n*=22), 8.8% (*n*=41) and 1.71% (*n*=8), respectively. The incidence of infection during VDLD induction in the SR and IR groups were 11.8 and 10.5% (*p=0.28*), respectively. The incidence of infection during and after CAM and before end of neutropenia in the SR and IR groups were 8.8 and 11.9% (*p=0.048*), respectively.

**Table 4:** The 5-year and 8-year overall survival rates, event free survival rates, cumulated incidence of relapse and 2-year therapy-related mortality for each groups

	SR groups	IR groups	HR groups	*p*
5-year OS (%)	90.7±1.4	86.0±1.2	68.5±2.5	*< 0.01*
8-year OS (%)	89.6±1.6	85.8±1.3	68.5±2.5	*< 0.01*
5-year EFS (%)	83.1±1.8	77.6±1.5	61.6±2.6	*< 0.01*
8-year EFS (%)	81.6±2.0	76.3±1.6	61.6±2.6	*< 0.01*
5-year CIR (%)	15.1±1.8	19.1±1.5	26.7±2.5	*< 0.01*
8-year CIR (%)	16.4±1.9	20.1±1.5	26.7±2.5	*< 0.01*
2-yearTRM (%)	1.7±0.6	3.9±0.7	12.7±1.8	*< 0.01*

Abbreviations: *OS* overall survival, *EFS* event free survival, *CIR* cumulated incidence of relapse, *TRM* therapy related mortality, *SR* standard risk, *IR* intermediate risk, *HR* high risk

In the SR group, 74 (15.2%) patients had MRD evaluated on Day 15 and Day 33 of induction therapy. Among them, 7 patients (9.46%) were MRD Day 33 positive (≥ 0.01%). When MRD detected patients were excluded, the 5-year OS and EFS of SR group were 90.1 ±1.5% and 82.6± 1.9%, respectively. When MRD Day 15 was analyzed, 34 patients (52%) were MRD Day 15 positive (≥ 0.1%). However, the OS, EFS, RFS were not significantly different between the MRD Day 15 positive group and the MRD Day 15 negative group. The 5-year OS and EFS of the MRD Day33 positive group (*n*=7) and the MRD Day33 negative group (< 0.01%) (*n*=67) were not significantly different. The incidence of relapse in the two groups was 28.6 and 7.5%, respectively (*p=0.129*). The ETV6-RUNX1 positive without other mutations group presented the relapse incidence of 3.0% (2/66); the ETV6-RUNX1 negative group presented 15% (30/200); the rest cases who did not performed mutation detection presented 23.6% (36/152).

## Discussion

In this study, we evaluated the efficacy and safety of risk stratification treatment strategy of the GD-2008-ALL protocol for childhood ALL. The most important result of this study is that, in the first 5 years since diagnosis, 71.9% of patients remained disease free and 83.5% were alive, which was largely different from the data from the research carried out by previous organized cooperative groups or single institutions in mainland China. Undoubtedly, the collaborative effort provided a major improvement in the ability to manage contemporary, intensive, and effective chemotherapy regimen for childhood ALL [3, 5, 13, 21, 22]. Over the last two decades, the world’s leading leukemia groups have achieved 5-year survival rates of approximately 90%, with 2 to 3% deaths as a result of toxicity in childhood ALL [13, 21, 22]. Since the 1990s, pediatricians in mainland China started the intensive chemotherapy for childhood ALL with the help from hematologists around the world, especially from those in Hong Kong where BFM ALL protocol was popular. This is why we modified our protocol according to the ALL-IC-BFM 2002 backbone therapy. In this collaborative protocol, we modified the intensity based on the conclusion of the local experience in the GZ-2002 ALL study [12] and BFM backbone therapy [3, 5]. The risk classification criteria were similar in these protocols. We agree that BFM-type chemotherapy in the hands of less experienced groups with limited medical resources was the potential risk of excessive TRM in the GZ-2002 protocol, where we observed a 4.8% rate of TRM in the SR and IR groups [12]. We modified the duration of CAM induction therapy and the total dose of CTX, Ara-c and 6-MP, resulting in the decrease of chemotherapy related mortality and toxicity in the SR and IR groups. In this study, we observed a 5.1±0.5% rate of TRM, ranging from 1.7±0.6% in the SR group, 3.9±0.7% in the IR group, and 12.7±1.8% in the HR group. GD-2008-ALL protocol successfully decreased the TRM and provided better OS rates. From 2002 to 2009, the multi-center collaborative clinical study of the GZ-2002 ALL protocol achieved 5-year EFS rates of 82.0±4.0% in the SR group and 78.0±5.0% in the IR group, respectively [12]. In this study, the 5-year EFS rates of the SR and IR groups were 81.5±1.8% and 75.3±1.5%, respectively. We believed that the 5-year EFS rates of the two protocols were similar when GD-2008-ALL protocol reduced the intensity of the SR group. Anyway, it contributed to the improvement of the OS rate of leukemic children in mainland China over the last 10 years.

Reduced dosage of CAM shortened the duration of neutropenia and resulted in a lower risk of severe infections. When we compared infection incidence during CAM therapy, the incidence of the IR group was significantly higher than the SR group. So, this was the primary reason for the decreased TRM. In previous published results from other centers, several studies focused on reducing intensity by giving fewer doses of anthracyclines and vincristine (VCR), like COG AALL 0331 regimen [13], DFCI ALL Protocol 05–001 [21] and Total Therapy XV regimen [22]. DCOG ALL 10 SR protocol [14] contains significantly less dose of anthracycline but more intensive first months of SR therapy than the UK ALL2003-SR protocol [23]. The Malaysia-Singapore 2003-SR protocol [15] contains slightly fewer anthracyclines (60 mg/m^2^) but more dexamethasone (560 mg/m^2^) and VCR (30 mg/m^2^). Graubner et al [24] and vanTiburg et al [25] showed that reduction of intensification significantly reduced infection rates. Even before the use of MRD measurement, good outcomes were achieved with relatively modest therapy as seen in a study among National Cancer Institute SR patients [26]. Reduced dosage of CAM has rarely been reported before. Our current report provided the real-world data for the safety and efficacy of this approach. For those without MRD detection available, our study illustrates that chemotherapy can be substantially reduced without jeopardizing outcomes in more than 70% of patients with ALL. SR patients have a 5-year OS rate of 90.1%, and received only nine intrathecal injections, a mild intensification and a traditional reinduction, followed by oral 6-MP/MTX maintenance. Relapses in SR usually occur late and three-quarters of these cases can be rescued. However, there is still room for improvement. It was reported lately in a Brazil study of very low risk ALL (similar to our SR but with Day 19 MRD < 0.01%) that EFS was excellent with 92% and OS with 96% [27]. COG AALL 0331 regimen [13] proved that the 6-year EFS of SR ALL can exceed 95%, that end-induction MRD of < 0.01% had a better outcome than MRD of 0.01 to < 0.1%, and that addition of intensified consolidation did not improve OS.

Since the technique of FCM-based MRD was not popular before 2012, included patients were stratified according to bone marrow morphology guidance of the GD-2008ALL protocol. Since 2012, the FCM detection of MRD has been available in several centers and the MRD was recorded without being recommended as criteria for risk stratification. Previous reports have proven the importance of MRD for the prognosis and risk stratification in childhood ALL [13]. Others have also shown that therapy reduction can be done safely in patients with favorable MRD [14, 15]. Previous studies from other centers have proven and we agree that MRD provided a powerful tool for evaluating early treatment response in ALL [28, 29]. Borowitz et al. showed that intensification of therapy in patients with moderately high MRD after induction delayed the occurrence of relapse [30], whereas another study showed that therapy intensification guided by MRD in the IR and HR cases contributed to lower relapse rates [14]. We also analyzed the MRD data of Day 15 and Day 33 and believe that, in this modified protocol for the SR group, stratification according to bone marrow morphology on Day 15 and Day 33 should be replaced by FCM-MRD. SR patients with detectable FCM-based MRD after Day 33 of reduction remission chemotherapy (MRD Day 33 positive) should not have accepted reduced intensification CAM therapy. The incidence of relapse in the subgroups of MRD Day 15 over 0.1% and below 0.1% was not significantly different, either was the incidence of relapse in the subgroups of MRD Day 15 over 0.01% and below 0.01%. Those with MRD Day 15 below 0.1% benefit from this protocol. The incidence of relapse in the subgroup of MRD Day 33 positive was higher than that in in the subgroup of MRD Day 33 negative. This result is similar to other collaborative centers’ reports. In the SR group, CIR was 16.4%, among which 7 cases should have been re-stratified into the IR group according to MRD detection by FCM. When stratified according to morphology, about 10% of the patients were unsuitable for reduced intensity induction. The long term data of follow-up have proven that the reduction of intensity of induction therapy did increase the risk of relapse when we did not stratify the SR and IR groups according to MRD level in induction. MRD low risk is believed to be related to increased OS and EFS. For MRD low risk patients, the VDLD2 and shortened CAM induction protocol guarantees both efficacy and safety.

ALL requires long-term standard treatment, and GD-2008-ALL-protocol has shown great therapeutic effects compared with historical studies. However, according to our center’s data, about 4.6% newly diagnosed childhood ALL patients, who were not included in this study, abandoned treatment without progression or recurrence for low expectation for the prognosis of childhood ALL. Reduced intensity of therapy means reduced cost of chemotherapy and of therapy for side effects, which is very important for families in developing countries. Meanwhile, reduced TRM would help develop the confidence of overcoming the disease in both parents and society. So, the social insurance policy in mainland China expanded the economic support budgets for children with ALL, which resulted in the decreased drop-out rate from 5.9% (58/976) during 2008 to 2012 to 2.9% (23/790) during 2013 to 2016 (*P* = 0.002).

## Conclusions

The SR group of the GD-2008-ALL protocol provided similar OS and EFS compared with historical BFM 2002 backbone therapy protocol while TRM was significantly lower than historical studies in China. The safety of reduction of intensity in the SR group is guaranteed. MRD based stratification is potentially practical in further reducing intensity in the SR group, especially in ETV6-RUNX1 ALL and MRD low risk SR group, which needs more study in the future.

## Supplementary Information

**Additional file 1.**

## Data Availability

The datasets used and/or analysed during the current study are available from the corresponding author on reasonable request.

## Abbreviations

ALL: Acute lymphoblastic leukemia
EFS: Event-free survival
MRD: Minimal residual disease
TRM: Therapy related mortality
BFM: Berlin-Frankfurt-Münster
SR: Standard risk
IR: Intermediate risk
HR: High risk
CTX: Cyclophosphamide
Ara-c: Cytarabine
6-MP: 6-mercaptopurine
MICM: Cell morphology, immunology, cytogenetics, and molecular biology
BM: Bone marrow
ISO: International Organization for Standardization
EGIL: Immunological Characterization of Leukemias
WHO: World Health Organization
FISH: Fluorescent in situ hybridization
CNS: Central nervous system
MTX: Methotrexate
PPR: Prednisone poor response
PGR: Prednisone good response
CR: Complete remission
CNSL: Central nervous system leukemia
IT: Intrathecal injection
HSCT: Hematopoietic stem-cell transplantation
MSD: Matched sibling donor
Ph+: Philadelphia chromosome-positive
OS: Overall survival
SE: Standard error
CIR: Cumulative incidence of relapse
VCR: Vincristine
