# Supplementary material for: Reduced intensity of early intensification does not increase the risk of relapse in children with standard risk acute lymphoblastic leukemia - a multi-centric clinical study of GD-2008-ALL protocol
Source: BMC Cancer. 2021 Jan 13;21:59. doi: 10.1186/s12885-020-07752-x (PMC7805214; doi:10.1186/s12885-020-07752-x)
Supplement: Supplementary file 1 — Additional file 1. [file 12885_2020_7752_MOESM1_ESM.docx]

Supplementary Information

**Reduced intensity of early intensification does not increase the risk of relapse in children with standard risk acute lymphoblastic leukemia - A multi-centric clinical study of GD-2008-ALL protocol**

Xin-Yu Li ^a,b^**,** Jia-Qiang Li ^a,b^, Xue-Qun Luo ^c^, Xue-Dong Wu ^d^, Xin Sun ^e^, Hong-Gui Xu ^a,b^ , Chang-Gang Li ^f^, Ri-Yang Liu ^g^, Xiao-Fei Sun ^h^, Hui-Qin Chen ^i^, Yu-Deng Lin ^j^, Chi-kong LI ^k^, Jian-Pei Fang ^a,b^.

**Author Affiliations:**

a. Department of Pediatrics, Sun Yat-sen Memorial Hospital, Sun Yat-sen University, Guangzhou 510120, China;

b. Guangdong Provincial Key Laboratory of Malignant Tumor Epigenetics and Gene Regulation, Sun Yat-sen Memorial Hospital, Sun Yat-sen University, Guangzhou 510120, China

c.The First Affiliated Hospital, Sun Yat-sen University, Guangzhou 510080, China;

d. Nanfang Hospital, Southern Medical University, Guangzhou 510515, China;

e. Guangzhou Women and Children’s Medical Center, Guangzhou 510623, China;

f. Shenzhen Children’s Hospital, Shenzhen 518038, China;

g. Huizhou Municipal Central People's Hospital, Huizhou 516001, China;

h. Sun Yat-sen University Cancer Center, Guangzhou 510060, China;

i. The Third Affiliated Hospital, Sun Yat-sen University, Guangzhou 510630, China;

j. Guangdong General Hospital, Guangzhou 510080, China;

k. Prince of Wales Hospital, The Chinese University of Hong Kong, Hong Kong 999077, China;

SI 1.

Immunity classification criteria

T lymphoblast (T-ALL) was identified by the positive T lymphocyte immune symbol: CD1, CD2, CD3, CD4, CD5, CD7, CD8 and TdT.

B lymphoblast (B-ALL) was identified by: ①early pre-B-ALL: HLA-DR positive or HLA-DR and CD19 both positive; ②common B lymphoblast: CD10 positive, HLA-DR, CD19 and CD22 positive, CyIg and SmIg negative, and divided into two subtypes: CD20 negative and CD20 positive; ③pre-B-lymphoblast: CyIg was positive, and most of the other B markers, like HLA-DR, CD19, CD22, CD10 and CD20 were positive; ④ mature B lymphocyte type: SmIg positive, CyIg positive/negative, CD19, CD22, CD20 and HLA-DR of other B immune markers were usually positive, with CD10 positive or negative.

SI 2.

Criteria for CNS involvement and CNS relapse

(1) Suspected CNSL (CNSL2)

Lymphoblasts are found in CSF, while cell count is no more than 5/μL, and RBC:WBC ratio is no more than 100:1, which indicates that the lumbar puncture is not traumatic, meaning that CSF contains no blood.

Lymphoblasts are found in CSF, while cell count is no more than 5/μL, and RBC:WBC ratio is over 100:1, which indicates that the lumbar puncture is traumatic, meaning that CSF contains blood.

The first lumbar puncture is bloody CSF without lymphoblast found, but the synchronous peripheral blood WBC is no less than 50 ×10^9^/L.

(2) Diagnosed CNSL (CNSL3)

CT/MRI shows massive brain lesions or meningeal infiltration.

Present of cranial nerve paralysis without other causes, even if CSF is negative or CT/MRI of the brain shows no abnormalities.

Retinopathy presents alone, even if no CSF lymphoblast or no MRI/CT brain lesion.

Lymphoblasts are found in CSF while WBC is no more than 5/μL and lumbar puncture is non-traumatic.

Massive lymphoblast is found in CSF, while WBC is more than 5/μL and RBC:WBC ratio is no more than 100:1.

Lymphoblast ratio in CSF is higher than peripheral blood, while WBC is more than 5/μL.

SI 3.

Details of intrathecal injection dose at different ages

Table S1 Intrathecal injection dose at different ages

| Age (months) | MTX (mg) | Ara-C (mg) | Dex (mg) |
| --- | --- | --- | --- |
| <12  ～23  ～35  ≥36 | 6  8  10  12 | 12  15  25  30 | 2  2  5  5 |

Note: for HR, T-ALL and central nervous system leukemia patients, MTX+Ara-C+Dex intrathecal injection was used. SR and IR BCP-ALL were intrathecal injected with MTX+Dex.

Abbreviations: MTX, methotrexate; Ara-C, cytarabine; DEX, dexamethasone.

SI 4.

Detail of cumulated dose of medicine in induction and reinduction therapy

Table S2 Cumulated dose of medicine in induction and reinduction therapy

|  | SR | IR | HR |
| --- | --- | --- | --- |
| VCR [1.5 mg/(m^2^·d)] | 8 | 8 | 8 |
| DNR [30 mg/(m^2^·d)] | 2 | 4 | 4 |
| DOX [30mg/(m^2^·d)] | 4 | 4 | 25 mg/(m^2^·d) X 4 |
| CTX [1000 mg/(m^2^·d)] | 2 | 3 | 3 |
| 6-MP [60 mg/(m^2^·d)] | 28 | 52 | 52 |
| Ara-C [75 mg/(m^2^·d)] | 16 | 24 | 24 |

Abbreviations: VCR, Vincristine; DNR, Daunorubicin; DOX, Adriamycin; CTX, cyclophosphamide; 6-MP, 6-mercaptopurine; Ara-C, cytarabine.
